# Effect of Toki-Shakuyaku-San on Regional Cerebral Blood Flow in Patients with Mild Cognitive Impairment and Alzheimer's Disease

**DOI:** 10.1155/2012/245091

**Published:** 2012-02-12

**Authors:** Teruyuki Matsuoka, Jin Narumoto, Keisuke Shibata, Aiko Okamura, Shogo Taniguchi, Yurinosuke Kitabayashi, Kenji Fukui

**Affiliations:** ^1^Department of Psychiatry, Graduate School of Medical Science, Kyoto Prefectural University of Medicine, 465 Kajii-cho, Kawaramachi-Hirokoji, Kamigyo-ku, Kyoto 602-8566, Japan; ^2^Department of Psychiatry, Gojouyama Hospital, 4-6-3 Rokujo-Nishi, Nara 630-8044, Japan

## Abstract

The aim of this study was to examine the effect of toki-shakuyaku-san (TSS) on mild cognitive impairment (MCI) and Alzheimer's disease (AD) using single-photon emission computed tomography (SPECT). All subjects were administered TSS (7.5 g/day) for eight weeks. SPECT and evaluations using the Mini Mental State Examination (MMSE), Neuropsychiatric Inventory, and Physical Self-Maintenance Scale were performed before and after treatment with TSS. Three patients with MCI and five patients with AD completed the study. No adverse events occurred during the study period. After treatment with TSS, regional cerebral blood flow (rCBF) in the posterior cingulate was significantly higher than that before treatment. No brain region showed a significant decrease in rCBF. TSS treatment also tended to improve the score for orientation to place on the MMSE. These results suggest that TSS could be useful for treatment of MCI and AD.

## 1. Introduction

Cholinesterase inhibitors (ChEIs) and memantine, an N-methyl-D-aspartate antagonist, are commonly used in treatment of Alzheimer's disease (AD). These drugs slow the progression of the disease and improve cognition, function, and behavior impairment but often have to be discontinued because of adverse events [1, 2]. There are no approved drugs for treatment of mild cognitive impairment (MCI). A systematic review showed that ChEIs were ineffective in preventing progression of MCI to AD or improving cognitive functions and led to adverse events [3].

There has been a recent increase in the use of traditional herbal medicine for treatment of dementia. Yokukan-san has been shown to improve behavioral and psychological symptoms of dementia (BPSD) [4, 5], hachimi-jio-gan improved cognitive function and activity of daily living (ADL) in AD [6], and choto-san was effective for patients with vascular dementia [7, 8]. Moreover, combination treatment with kami-untan-to and donepezil improved cognitive function and increased regional cerebral blood flow (rCBF) in the bilateral frontal lobes [9].

Toki-shakuyaku-san (TSS) is mainly used for gynecologic disorders but has also been used for treatment of cognitive impairment based on accumulated evidence of its neuroactive and neuroprotective effects. TSS activates cholinergic [10–14] and monoaminergic [12, 13, 15] neurons and has a protective effect on amyloid *β* [16, 17], an antioxidant effect [18, 19], and an antiapoptosis action [12]. Several clinical studies have also suggested that TSS improves cognitive impairment in dementia, MCI, and poststroke patients [20–22]. Brain imaging may be useful to examine the effects of TSS. Therefore, the aim of this study was to identify the effects of TSS in patients with MCI and AD using single-photon emission computed tomography (SPECT).

## 2. Methods

### 2.1. Subjects

The subjects were 13 patients treated at the Center for Diagnosis of Dementia at the Kyoto Prefectural University of Medicine. Four patients were diagnosed with MCI and 9 with AD, based on Petersen et al. [23] and the National Institute of Neurological and Communicative Disease and Stroke-Alzheimer's Disease and Related Disorders Association (NINCDS-ADRDA) criteria for probable AD [24]. We excluded patients with a significant history of psychiatric or neurological disorders (other than MCI and AD), including stroke, head injury, epilepsy, psychiatric disorders, alcohol abuse, or a serious medical condition. Participants had not been prescribed ChEIs or memantine since our center examines patients who have not been diagnosed with dementia. This study was approved by the Ethics Committee of the Kyoto Prefectural University of Medicine. Informed consent was obtained from all of the patients.

### 2.2. Study Protocol

Subjects were treated with TSS given as a daily dose of 7.5 g of powder for eight weeks. During this period, new medications were not introduced. TSS is registered in the Pharmacopoeia of Japan as Kampo Medicine TJ-23. The TSS used in the study was provided by Tsumura (Tokyo, Japan) and was prepared from the extract of a mixture of dried plants: 4.0 g Paeoniae radix, 4.0 g Atractylodis lanceae rhizoma, 4.0 g Alismatis rhizoma, 4.0 g Hoelen, 3.0 g Cnidii rhizome, and 3.0 g Angelicae radix. All subjects underwent magnetic resonance imaging (MRI) or computed tomography (CT) before treatment. SPECT was performed before and after treatment.

Cognitive impairment was evaluated using the Mini Mental State Examination (MMSE) [25] before and after the study period. The MMSE has 11 subscales including orientation to time, orientation to place, registration, attention, recall, naming, repetition, auditory comprehension/command, reading comprehension, sentence construction, and constructional praxis. BPSD were evaluated before and after the study period using the Neuropsychiatric Inventory (NPI) [26]. The NPI is a caregiver-based clinical instrument that evaluates 10 domains of neuropsychiatric symptoms in dementia: delusions, hallucinations, agitation, depression, anxiety, euphoria, apathy, disinhibition, irritability, and aberrant motor behavior. The frequency score ranges from 0 to 4 points, and the severity score ranges from 0 to 3 points. The NPI score for each subscale is created by multiplying the frequency and severity scores, with a maximum score of 12. Therefore, the NPI total score ranges from 0 to 120. Higher scores denote a greater severity of a symptom. ADL before and after the study period were evaluated using the Physical Self-Maintenance Scale (PSMS) [27], which consists of 6 items related to physical activities: toileting, feeding, dressing, grooming, ambulating, and bathing. A lower total score indicates greater impairment of ADL.

### 2.3. Image Acquisition and Analysis

Brain perfusion SPECT was performed by intravenous injection of 185 MBq of N-isopropyl-p-[^123^I]iodoamphetamine (I-123-IMP) (Nihon Mediphysics, Hyogo, Japan) in subjects seated at rest with their eyes open. SPECT imaging commenced 22 min after the injection and continued for 16 min. A triple-head gamma camera (Prism Irix, Picker International, Cleveland, OH, USA) and a low-energy, high-resolution, and parallel collimator were used. Projection data from each camera were obtained in a 128 × 128 format for 40 angles of 1201 at 8 s per angle (voxel size: 2 × 2 × 2 mm).

Image analysis was performed using Statistical Parametric Mapping (SPM) 8 (Wellcome Department of Cognitive Neurology, University College, London, UK) in Matlab 7.5 (Mathworks Inv., Sherborn, MA, USA). After confirmation of no significant artifacts due to atrophy using MRI or CT scans, all SPECT images were anatomically normalized using the I-123-IMP template (Fujifilm RI Pharma, Tokyo, Japan) matched to the Montreal Neurological Institute (MNI) template. The normalized images were smoothed using a 12-mm full-width half-maximum (FWHM) isotropic Gaussian kernel. To examine regional differences, the images were scaled to a mean global cerebral blood flow of 50 mL/100 g/min. 

### 2.4. Statistical Analysis

A Wilcoxon signed rank test was used to analyze the changes in MMSE, NPI, and PSMS scores. Data were analyzed using SPSS 12.0 J for Windows (SPSS Inc., Chicago, IL, USA). *P* < 0.05 was considered statistically significant. A paired *t*-test was performed to determine whether TSS affected rCBF in patients with MCI and AD. The *X*, *Y*, and *Z* coordinates provided by SPM approximate the MNI brain space. The statistical thresholds were set to a family-wise error (FWE)-corrected *P* value of 0.05 at the voxel level.

## 3. Results

### 3.1. Subject Characteristics

Eight of the 13 subjects completed the study. Five were discontinued because of poor compliance (*n* = 3), a move to another area (*n* = 1), and refusal to continue (*n* = 1). None of the subjects had adverse events. The characteristics of the 8 subjects who completed the study are shown in Table 1. Six had not been prescribed with a psychoactive drug, 1 had taken risperidone (1 mg/day), and 1 had taken rilmazafone (0.5 mg/day) before the start of the study. Both subjects continued to take these drugs during the study.

### 3.2. Changes in MMSE, NPI, and PSMS Scores

Changes in MMSE, NPI, and PSMS scores are shown in Table 2. At baseline, cognitive impairment and BPSD were mild, and ADL was high. Scores for the MMSE, NPI total, and PSMS did not change significantly after TSS treatment. Among the MMSE subscales, the score for orientation to place showed a tendency to improve (*P* = 0.025), but the change was not significant using a Bonferroni correction (*P* = 0.025 > 0.05/11) (Table 3).

### 3.3. Paired *t*-Test Using SPM

**Figure 1:**
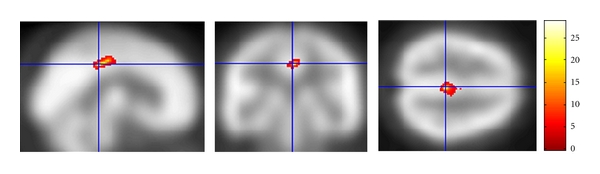
Regions showing a significant increase in rCBF after TSS treatment. The statistical thresholds were set to an uncorrected *P* value of 0.001 at the voxel level and to a corrected *P* value of 0.05 at the cluster level.

The paired *t*-test showed a significant increase in rCBF in the posterior cingulate after TSS treatment compared to before treatment (Table 4). When the statistical thresholds were set to an uncorrected *P* value of 0.001 at the voxel level and a corrected *P* value of 0.05 at the cluster level, the posterior cingulate was the only brain region that showed a significant increase in rCBF (Figure 1). No brain region showed a significant decrease in rCBF.

## 4. Discussion

In this study, rCBF in the posterior cingulate was significantly increased after eight weeks of treatment with TSS. The MMSE, NPI total, and PSMS scores did not worsen and adverse events did not occur during the treatment. Moreover, TSS treatment tended to improve the score for orientation to place on the MMSE.

Some clinical studies have demonstrated improvement in cognitive impairment by TSS in dementia and poststroke patients. Inanaga et al. reported improvement in orientation to time and place, spontaneous activity, emotional lability, and motivation in 80 dementia patients (40 with vascular dementia, 38 with AD, and 2 with mixed dementia) after 12 weeks of TSS treatment [20], while Goto et al. demonstrated that TSS is effective in suppressing impairment of visuospatial perception and the lower limbs in poststroke patients [22]. In our subjects, orientation to place tended to improve while other cognitive impairments and BPSD were unchanged. These findings are partly consistent with previous studies and suggest that TSS might be particularly effective for improvement of spatial perception.

TSS treatment in this study was associated with a change in rCBF in the posterior cingulate. This brain region plays an important role in many cognitive functions, including visuospatial orientation, topokinesis, navigation of the body in space, self reflection, autobiographical memory, and assessment of objects in space in terms of first-person orientation [28]. Connections between the posterior cingulate and the parahippocampus are likely to play a major role in memory-related functions [29], and these connections may be disturbed in MCI and AD [30, 31]. Moreover, hypofunction in the posterior cingulate is associated with visuoperceptual deficits in MCI and AD [32], as well as disorientation to time and place in AD [33]. In this study, TSS increased rCBF in the posterior cingulate and improved orientation to place based on the MMSE. Therefore, TSS might be useful for treatment of cognitive impairment associated with the posterior cingulate.

Previous studies have shown that donepezil increases or maintains rCBF, mainly in the frontal lobes [34–37]. In contrast, in this study, TSS increased rCBF in the posterior cingulate. In addition to the cholinergic effect, TSS also has monoaminergic, antiamyloid, antioxidant and antiapoptosis effects [10–19]. Collectively, these results suggest that TSS has different effects on cognitive impairment compared to those of donepezil. Therefore, combining TSS with donepezil might have an additional benefit in treatment of MCI and AD.

The study has several limitations, since it was performed with an open label design with no control group, the observation period was short, and the sample size was small. These limitations may reduce the strength of the results, but the findings were partly consistent with previous studies. Moreover, to our knowledge, this is the first study to demonstrate an effect of TSS on rCBF in patients with MCI and AD.

## 5. Conclusions

Treatment with TSS significantly increased rCBF in the posterior cingulate and tended to improve orientation to place in MCI and AD patients. Therefore, TSS might be useful for treatment of MCI and AD. Moreover, since the effects of TSS on rCBF may differ from those of donepezil, combination therapy of TSS and donepezil might be particularly effective. A study in a large number of MCI and AD patients is needed to confirm the effects of TSS.

## Figures and Tables

**Table 1:** Clinical characteristics of subjects who completed the study.

Item	Value
Sex, M/F	3/5
Handedness, R/L	8/0
Age, y.o.	77.8 ± 4.9
Diagnosis, MCI/AD	3/5
Age at onset, y.o.	76.3 ± 4.3
Duration of illness, years	1.6 ± 1.6
Education, years	12.0 ± 1.7

AD: Alzheimer's disease; F: female; L: left; M: male; MCI: mild cognitive impairment; R: right; SD: standard deviation; y.o.: years old.

Values are shown as a number ratio or as the mean ± SD.

**Table 2:** Changes in MMSE, NPI and PSMS scores from before to after TSS treatment for 8 weeks.

	Baseline mean ± SD	8 weeks mean ± SD	*P* value
MMSE	23.4 ± 3.6	23.9 ± 3.8	0.279
NPI total score	5.5 ± 5.9	3.9 ± 4.6	0.180
PSMS	5.9 ± 0.4	5.4 ± 1.4	0.414

MMSE: Mini Mental State Examination; NPI: Neuropsychiatric Inventory; PSMS: Physical Self-Maintenance Scale; SD: standard deviation.

**Table 3:** Changes in MMSE subscale scores from before to after TSS treatment for 8 weeks.

	Baseline mean ± SD	8 weeks mean ± SD	*P* value
Orientation to time	3.5 ± 1.6	3.1 ± 2.1	0.414
Orientation to place	3.9 ± 0.4	4.5 ± 0.5	0.025
Registration	3.0 ± 0.0	3.0 ± 0.0	1.000
Attention	3.5 ± 1.6	3.4 ± 1.6	0.655
Recall	1.0 ± 1.2	1.0 ± 0.9	0.891
Naming	2.0 ± 0.0	2.0 ± 0.0	1.000
Repetition	0.9 ± 0.4	1.0 ± 0.0	0.317
Auditory comprehension/command	2.8 ± 0.5	3.0 ± 0.0	0.157
Reading comprehension	1.0 ± 0.0	1.0 ± 0.0	1.000
Sentence construction	1.0 ± 0.0	1.0 ± 0.0	1.000
Constructional praxis	0.9 ± 0.4	0.9 ± 0.4	1.000

MMSE: Mini Mental State Examination; SD: standard deviation.

**Table 4:** Results of paired *t*-tests.

Brain area	MNI coordinates at the center of the cluster			*Z* value at the local maximum	Voxel *P* value (corrected)	Cluster size	Cluster *P* value (corrected)
	*X*	*Y*	*Z*				
Posterior cingulate	2	−32	40	5.66	0.002	6	<0.001
	4	−24	44	5.08	0.039	1	0.015
